# Supplementary material for: An inversion problem for optical spectrum data via physics-guided machine learning
Source: Sci Rep. 2024 Apr 19;14:9042. doi: 10.1038/s41598-024-59594-3 (PMC11031606; doi:10.1038/s41598-024-59594-3)
Supplement: Supplementary file 1 — Supplementary Information. [file 41598_2024_59594_MOESM1_ESM.pdf]

Supplemental Material
An inversion problem for optical spectrum data via physics-guided machine learning

Hwiwoo Park[1], Jun H. Park[2,†], and Jungseek Hwang[1,*]

[1]*Department of Physics, Sungkyunkwan University, Suwon, Gyeonggi-do 16419, Republic of Korea*

[2]*School of Mechanical Engineering, Sungkyunkwan University, Suwon, Gyeonggi-do 16419, Republic of Korea*

Appendix A: Model architectures

In our comparative analysis presented in the main text, we evaluate three models: FCN, CNN, and rRIM. Here's a concise overview of their architectures.

1. FCN (Fully Connected Network)

- Input and output layers contain 501 and 3001 neurons, respectively. These layer configurations remain consistent across all models.
- Three hidden layers with 1024, 2048, and 1024 neurons.
- ReLU activations are applied to all layers.
- A dropout with a probability of 0.2 is implemented in the last hidden layer.

2. CNN (Convolutional Neural Network)

- Incorporates two one-dimensional convolutions following the input layer.
- A fully connected layer connects the convolutional layers to the output layer.
- All layers utilize ReLU activations.
- The initial 1D convolutional layer expands the channel from 1 to 16, and the subsequent layer further expands it to 32.
- Both convolutional layers use kernel sizes of 53 and 33.

3. rRIM (regularized Recurrent Inference Machine)

- The update network, denoted as m_ϕ, comprises a stack of three GRU cells and an output layer. Here is the update equation for reference,

$$x_{t+1} = x_t + g_t,$$

$$\begin{bmatrix} g_t \\ s_{t+1} \end{bmatrix} = m_\phi(\nabla_t, s_t),$$

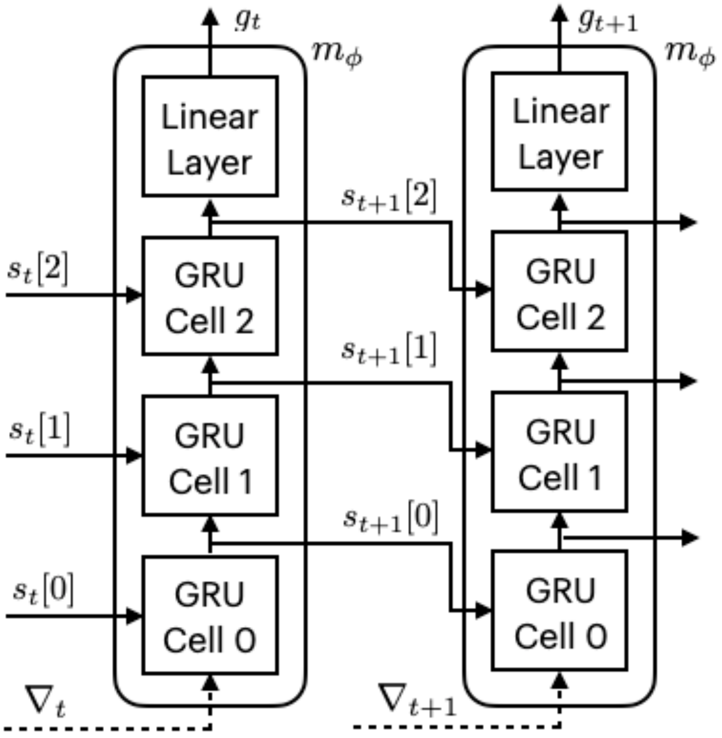

Figure S1. A schematic diagram of a stacked GRU in the update network.

where s_t represents a latent memory variable.

- Each GRU cell contains 1024 neurons.
- The gradient of the current state, x_t, is initially introduced into the first cell and subsequently propagated through the remaining GRU cells for each time step along with memory variables.
- The output, g_t, from these stacked GRU units is combined with the input, x_t to yield x_{t+1} which is then used to calculate the gradient in the next step.
- Memory variables from each GRU unit are passed to the corresponding GRU units in the next time step, as illustrated in Fig. S1.
- Notably, for training, the gradient obtained from the loss function in Eq. (10) in the main text only flows through the solid edges, as explained in the main text.

Appendix B: Generation of training data

The training data are generated based on existing experimental results [1, 2]. A mixture of Gaussians is used as a model for the paring glue function as follow

$$I^2\chi(\omega, T) = \sum_{i=1}^{N_G} \frac{A_{p_i}}{\sqrt{2\pi}(d_i/2.35)} \exp\left\{-\frac{(\omega-\omega_{p_i})^2}{2(d_i/2.35)^2}\right\},$$

where N_G is a number of Gaussians and A_{p_i}, ω_{p_i}, and d_i represent the amplitude, location and width of the i-th Gaussian peak, respectively. These parameters are sampled from uniform distributions with the specified ranges: $N_G \in \{1, 2, 3, 4\}$, $A_{p_i} \in [1, 300]$ meV, $w_{p_i} \in [40, 250]$ meV, and $d_i \in [140, 360]$ meV. Note that the dependence of A_{p_i}, ω_{p_i}, and d_i on temperature T in Kelvin is suppressed for notational simplicity. To generate the corresponding optical scattering rate $1/\tau^{\rm op}(\omega)$, we discretize the following generalized Allen formula [1–4]

$$\frac{1}{\tau^{\rm op}(\omega, T)} = \int_0^\infty d\Omega\, I^2\chi(\Omega, T)\, K(\omega, \Omega, T), \tag{S1}$$

with the Shulga's kernel [4]

$$\begin{aligned} K(\omega, \Omega, T) =& \frac{\pi}{\omega}\left[2\omega \coth\left(\frac{\Omega}{2T}\right) - (\omega+\Omega)\coth\left(\frac{\omega+\Omega}{2T}\right)\right. \\ &\left. +(\omega-\Omega)\coth\left(\frac{\omega-\Omega}{2T}\right)\right] \end{aligned}$$

using the Simpson's rule [5]. We truncate the integral in Eq. (S1) at 500 as the values of $I^2\chi$ become 0 long before this point. We discretize Ω and ω into 3000 and 500 points, respectively. We verified that the discretized version was in agreement with the continuous version.

Samples of glue functions and their corresponding optical scattering rates with different noise levels are depicted in Fig. S2.

Appendix C: Equivalence between rRIM and iterative Tikhonov regularization

In this section, we derive the gradient formula presented in the main text,

$$\nabla_t = \frac{1}{\sigma^2} A^T(y - Ax_t)$$

and show the equivalence between rRIM and iterative Tikhonov regularization. From Eq. (5) in the main text and the assumption of the normally distributed noise, we have

$$y - Ax \sim N(0, \sigma^2 I), x \in \mathbb{R}^n,$$

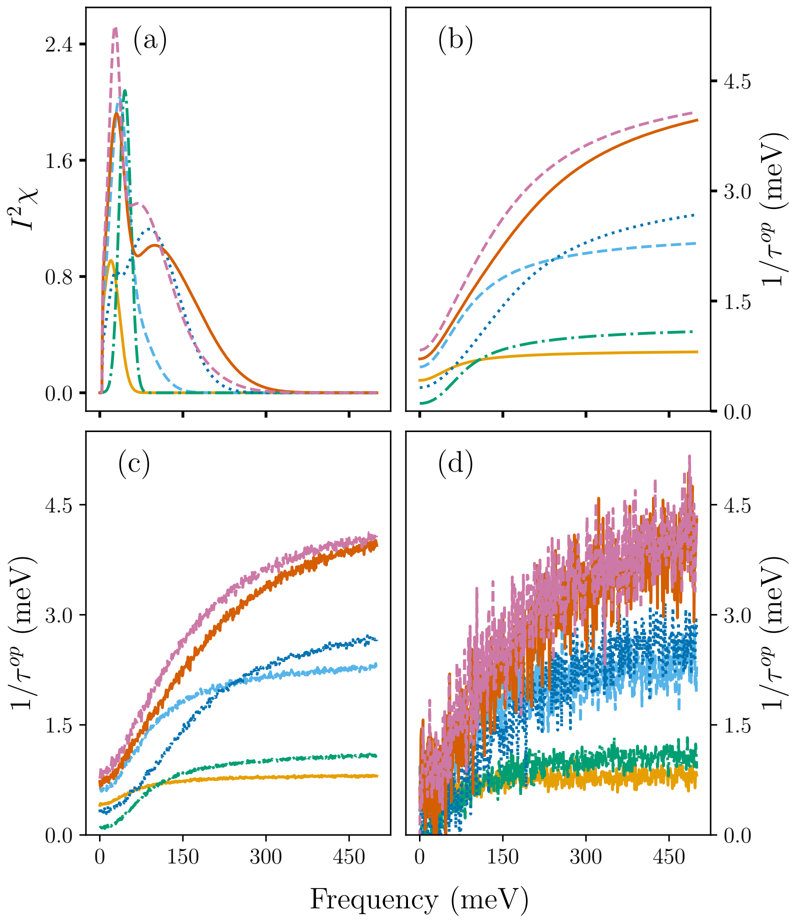

Figure S2. (a) Examples of $I^2\chi$ in the training data and their corresponding $1/\tau^{\mathrm{op}}$ with noise levels (b) $\sigma = 0$, (c) $\sigma = 0.001$, and (d) $\sigma = 0.1$. (All $1/\tau^{\mathrm{op}}$'s are scaled down by 300).

which can be written as a conditional probability density

$$p(y|x) = \frac{1}{(2\pi)^{n/2}\sigma} \exp\left\{-\frac{1}{2\sigma^2}(y - Ax)^T(y - Ax)\right\}.$$

The log probability becomes

$$\log p(y|x) = -\frac{1}{2\sigma^2}(y - Ax)^T(y - Ax) + C,$$

where C is a constant. Finally, taking the gradient, we get

$$\nabla_x \log p(y|x) = \frac{1}{\sigma^2}A^T(y - Ax).$$

The gradient based recursive algorithm in Eq. (7) from the main text can be expressed as

$$x_{t+1} = x_t + \beta_t A^T(y - Ax_t), \tag{S1}$$

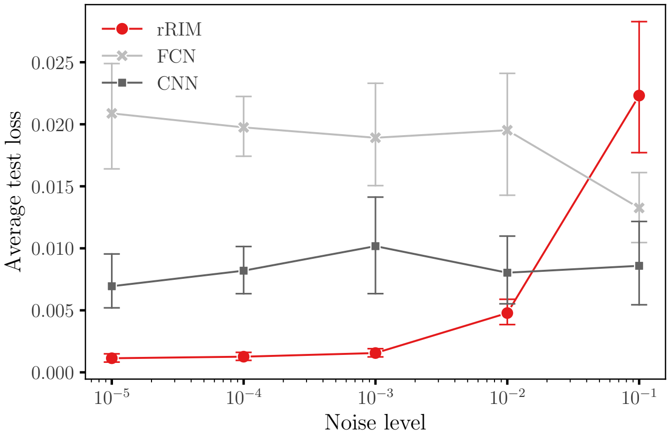

(a)

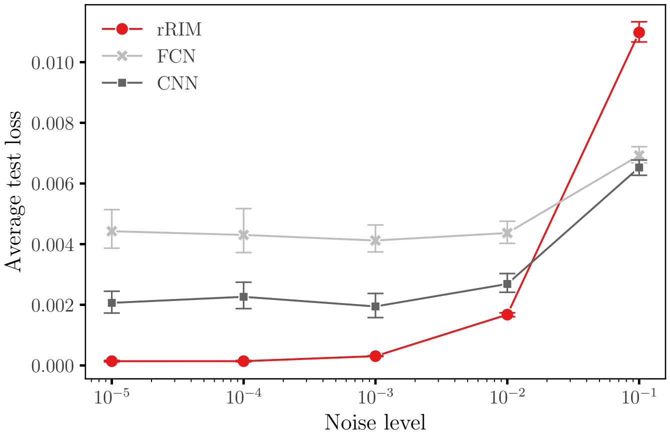

(b)

Figure S3. Comparison of average test losses for rRIM, FCN, and CNN with different noise levels for data set sizes of (a) $N = 100$ and (b) $N = 10000$.

where $\beta_t = \frac{\gamma_t}{\sigma^2}$. Comparing with Eq. (7) of the main text, we note that the the gradient of log prior, $\nabla \log p(x)$, is missing, which is implicitly restored in the RIM formulation as explained in the main text.

To show the equivalence, we start from the normal equation

$$A^T A x = A^T y$$

from which we can derive an iterative method,

$$x_{t+1} = x_t + \beta A^T (y - A x_t). \tag{S2}$$

This iterative approach is known as the Landweber iteration. The equivalence between

RIM and Landweber iteration arises from the similarity between Eqs. (S1) and (S2). Thus, rRIM can be regarded as an effective and efficient optimizer for solving iterative Tikhonov regularization.

It is important to note that the Landweber iteration is impractical due to its slow convergence property. To address this, we adopt the preconditioned Landweber iteration, represented as

$$x_{t+1} = x_t + \beta D A^T (y - A x_t). \tag{S3}$$

Here, the preconditioning factor is set as $D = (A^T A + h^2 I)^{-1}$ [6].

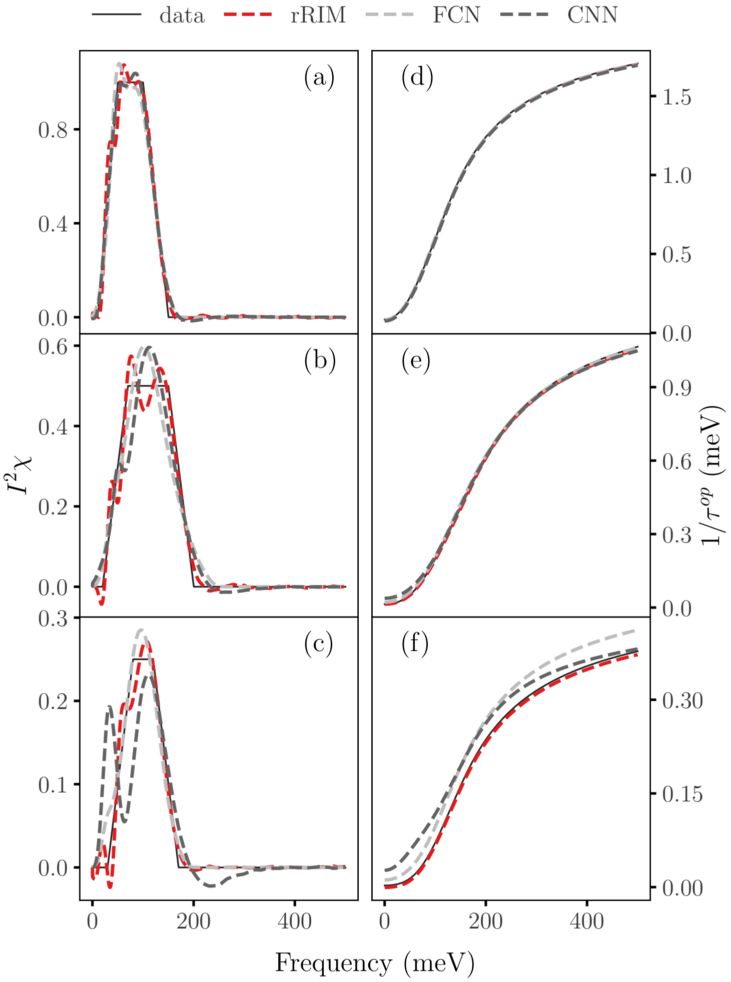

Figure S4. (Color online) Comparison of prediction capabilities of rRIM, FCN, and CNN for trapezoidal OOD data samples: (a), (b), and (c) and their corresponding $1/\tau^{op}$'s: (d), (e), and (f).

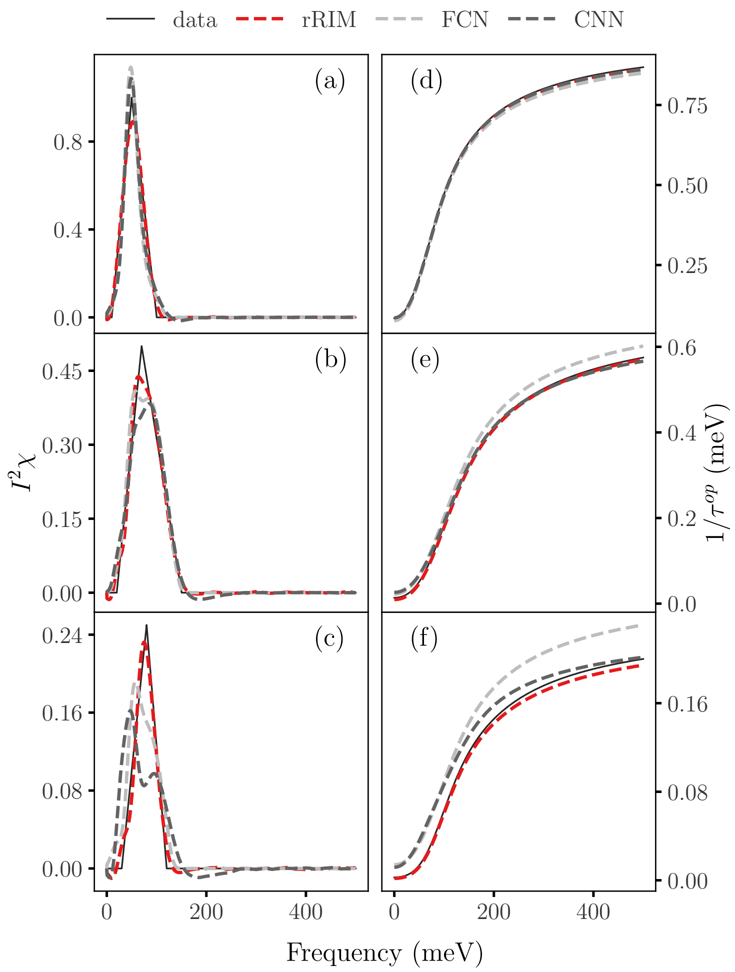

Figure S5. Comparison of prediction capabilities of rRIM, FCN, and CNN for triangular OOD data samples: (a), (b), and (c) and their corresponding $1/\tau^{op}$'s: (d), (e), and (f).

Appendix D: Noise robustness of rRIM

As discussed in the main text, rRIM is robust to noise, regardless of the data set sizes. In Fig. S3, we present the average test losses of rRIM, FCN, and CNN for varying noise amplitudes with data set sizes of $N = 1000$ and $N = 10000$. The results show that rRIM outperforms the other models up to a specific noise level. However, regardless of the sample sizes, the ill-posedness nature of the problem becomes evident when the noise level exceeds a certain threshold.

Appendix E: Handling OOD data capacity of rRIM

To illustrate rRIM's capability to handle OOD data, we apply rRIM to trapezoidal and triangular waves with varying widths and heights. These results are compared with those obtained from FCN and CNN in Figs. S4 and S5. Notably, we observe that rRIM outperforms FCN and CNN as the given data deviated further from the scope of the training dataset. This superiority is also evident in the corresponding $1/\tau^{op}$'s, which exhibits similar patterns.

---

[1] E. Schachinger, D. Neuber, and J. P. Carbotte, Phys. Rev. B **73**, 184507 (2006).

[2] J. Hwang, T. Timusk, E. Schachinger, and J. P. Carbotte, Phys. Rev. B **75**, 144508 (2007).

[3] P. B. Allen, Phys. Rev. B **3**, 305 (1971).

[4] S. V. Shulga, O. V. Dolgov, and E. G. Maksimov, Phys. C Supercond. its Appl. **178**, 266 (1991).

[5] W. H. Press, S. A. Teukolsky, W. T. Vetterling, and B. P. Flannery, *Numerical Recipes 3rd Edition: The Art of Scientific Computing*, 3rd ed. (Cambridge University Press, 2007).

[6] A. Neumaier, SIAM Review **40**, 636 (1998).
